# Influence of Low TiO_2_ Nanoparticle Loading on Electrospun sPEEK Fiber Morphology and Chemistry and on the Tensile Properties of Corresponding Solution-Cast Membranes

**DOI:** 10.3390/polym18192410

**Published:** 2026-10-02

**Authors:** Dayana Llumiquinga, Luis Criollo, Wilson Navas-Pinto, Germán Omar Barrionuevo, Jhon Luna-Jaén, Xavier Sánchez-Sánchez, Santiago D. Puma-Araujo

**Affiliations:** 1 Departamento de Ciencias de la Energía y Mecánica, Universidad de las Fuerzas Armadas ESPE, Sangolquí 171103, Ecuador; dlllumiquinga@espe.edu.ec (D.L.); lmcriollo@espe.edu.ec (L.C.); wmnavas1@espe.edu.ec (W.N.-P.); jaluna3@espe.edu.ec (J.L.-J.); 2 Centro de Nanociencia y Nanotecnología, Universidad de las Fuerzas Armadas ESPE, Sangolquí 171103, Ecuador; 3 Department of Mechanical Engineering, Universidad San Francisco de Quito USFQ, Quito 170901, Ecuador; gbarrionuevo@usfq.edu.ec; 4 School of Engineering and Sciences, Tecnologico de Monterrey, Monterrey 64849, Mexico

**Keywords:** polymer matrix composites, electrospinning, sPEEK, TiO_2_, nanoparticle loading, mechanical properties

## Abstract

Polymer matrix composites (PMCs) have garnered importance due to their unique combination of mechanical, thermal, and functional properties. However, their performance depends on overcoming challenges related to dispersion, bonding, and processing. Electrospinning is an advanced technique for producing nanofiber or microfiber PMCs. One of the key challenges in electrospun PMCs is the uniform dispersion of reinforcement materials like ${\mathrm{TiO}}_{2}$ nanoparticles within the polymer matrix. A Taguchi orthogonal design ($L_{16}$) was used to evaluate the effects of needle-to-collector distance, applied voltage, and flow rate on mean fiber diameter and deposition quality. Scanning electron microscopy (SEM) and Fourier-transform infrared spectroscopy (FT-IR) were used to characterize electrospun neat sulfonated poly(ether ether ketone) (sPEEK) and sPEEK/TiO_2_ fibers, whereas tensile tests were performed on solution-cast membranes prepared from the corresponding formulations. The lowest observed run mean diameter was 204.6695 nm at 8 kV, 1.25 mL h^−1^, and a needle-to-collector distance of 12 cm; this run was also selected for deposition quality. Neat sPEEK membranes exhibited a mean tensile strength of 35.3 MPa, a mean apparent tensile modulus of 476 MPa, and a mean elongation at fracture of 18%. Among the TiO_2_-containing formulations, increasing the TiO_2_ content from 0.04 to 0.10 wt.% was associated with higher mean apparent tensile modulus and tensile strength and lower mean elongation at fracture. The 0.10 wt.% formulation exhibited the highest mean apparent tensile modulus and tensile strength.

## 1. Introduction

Electrospinning has drawn attention from the scientific community due to its versatility in producing fibers with diameters ranging from a few nanometers to some micrometers by a relatively simple and straightforward process [1,2], which can be later used for applications in different fields, such as reinforced composites, biomedical applications, filtration, and protective clothing, among others [3]. This technique can be considered as a derivation of electrospraying, which was first developed by Nollet [4] and consists of creating a jet flow of a polymer solution or melt by applying high electrical forces [5]. Since then, different researchers have extensively worked on this method with outstanding results and breakthroughs [6,7,8,9,10,11,12].

Larrondo and Manley [7] used polyethylene and paraffin solutions to be jet-drawn by the application of high voltages and observed the morphology of the meniscus predicted by Taylor [6]. Doshi and Renecker [8] electrospun narrow fibers from an aqueous solution of polyethylene oxide (PEO) and observed an inverse relation between the distance from the jet tip and the collector and the diameter of the obtained fiber. Furthermore, Fong et al. [9] observed that lower fiber diameters are produced by modifying the polymer solution with different solvents and increasing the applied voltage. Shin et al. [10] observed that a lower fiber diameter can be achieved by stretching and accelerating the filament preceding its impact on the collector. By controlling processing parameters such as voltage, flow rate, needle-collector distance, and solution properties, suitable electrospun fibers with the desired diameter, morphology, and properties can be obtained.

Advancements in the understanding of the electrospinning process have enabled the electrospinning of more than 100 polymers, and different reinforcement particles have been added to those polymers to obtain novel materials that have been characterized through several techniques [11]. For instance, Casasola et al. [12] electrospun poly lactic acid (PLA) fibers with different solvent systems and observed that acetone/dimethylformamide produced the thinnest nanofibers without defects. Similarly, Kariduraganavar et al. [13] electrospun fibers of poly(ϵ-caprolactone) (PCL) with the presence of a mixture of dimethylformamide, dichloromethane, and dibenzylidene sorbitol as a solvent and successfully produced fibers with diameters around 600 nm. In the same way, Li et al. [14] successfully added silver nanoparticles to sulfonated polyether ether ketone (sPEEK) nanofibers through electrospinning with a concentration ranging from 5 to 15% and using a solution of dimethylformamide (DMF) as a solvent. The authors observed that silver particles could interact with sulfonated groups.

Within the different polymers used to manufacture fibers employing electrospinning, PEEK has shown to be effectively used in a wide range of temperatures at lower costs than traditional polymers [15]. PEEK has a lower cost and superior availability than other traditional polymers [16]. Moreover, PEEK exhibits acceptable tensile strength and elevated melting and degradation temperatures [17,18]. In order to improve the hydrophilicity of the polymer and its solubility in different solvents, a sulfonation process is performed to add sulfur-based groups in the polymer chains [19,20].

Due to the development of technology and the current more demanding applications, different reinforcement particles have been gradually added to polymers such as sPEEK to produce novel and more resistant materials with specific properties for tailored applications. Boaretti et al. [21] produced sPEEK nanofibers later reinforced with Aquivion, improving the mechanical strength of the composite materials compared to pure Aquivion. Likewise, Chamakh and Ayesh [17] produced sPEEK/PVP nanofibers reinforced with different weight percentages of ruthenium dioxide (${\mathrm{RuO}}_{2}$) and concluded that the electrical resistivity of the produced fibers decreased with increasing concentrations of the reinforcement. Jia et al. [22] developed sPEEK/poly(methyl methacrylate) fibers reinforced with carbon fiber particles at different weight percentages and concluded that the mechanical properties reached a maximum increase when 20 wt.% of the reinforcement was added to the polymer matrix composite (PMC).

Several nanostructured fillers have been incorporated into sPEEK-based systems to target different functional and mechanical requirements. SiO_2_-containing systems have been investigated to improve dimensional stability and membrane performance, whereas carbon nanotubes and sulfonated graphene oxide have been used to enhance mechanical, transport, and barrier properties. RuO_2_-containing sPEEK-based materials have mainly been investigated for modifying electrical behavior [17,23,24,25]. These reinforcements therefore address different performance requirements and cannot be ranked solely on the basis of a single property. TiO_2_ was selected for the present study because the literature reports a favorable combination of thermal and chemical stability, surface activity, and mechanical reinforcement in polymer-based composites [26,27]. In addition, the present work evaluates as-received TiO_2_ nanoparticles at very low contents, ranging from 0.04 to 0.10 wt.%, thereby limiting the amount of inorganic filler while examining its influence on the morphology, chemical structure, and mean mechanical response of the sPEEK/TiO_2_ system. These characteristics represent advantages relevant to the formulation investigated in this work; however, because alternative nanoparticles were not experimentally evaluated, no universal superiority of TiO_2_ over other nanofillers is claimed.

In the last decade, titanium dioxide (${\mathrm{TiO}}_{2}$) has gained importance as reinforcement in polymer-based composite materials due to its low reactivity, high corrosion resistance [28,29], adequate mechanical properties [26], great thermal stability [30,31], acceptable biocompatibility [32], high surface activity [33], good dispersibility [33], and potential photocatalytic activity [34], among other beneficial properties and possible future applications. Radhakrishnan et al. [28] developed PMCs with a polyaniline matrix reinforced with ${\mathrm{TiO}}_{2}$ nanoparticles and observed improved corrosion resistance of the composite compared to the matrix material. In the same way, Díez-Pascual and Díez-Vicente [26] produced ternary composite materials with a PEEK-poly(ether imide) (PEI) matrix and nano-${\mathrm{TiO}}_{2}$ reinforcement. The study concluded that the gradual addition of reinforcement increased the tensile properties, thermal stability, and flame-retardant capability of the PMC. Similarly, Nayak et al. [27] developed epoxy-based composites reinforced with glass fiber and ${\mathrm{TiO}}_{2}$ nanoparticles. The study concluded that adding 0.1 wt.% of ${\mathrm{TiO}}_{2}$ to a glass fiber-reinforced polymer modified the water diffusion coefficient, flexural strength, and residual interlaminar shear strength.

More recently, Navas-Pinto et al. [35] investigated bio-epoxy/glass fiber composites reinforced with ${\mathrm{TiO}}_{2}$ nanoparticles and reported improvements in tensile and flexural properties. In particular, the addition of ${\mathrm{TiO}}_{2}$ produced improvements of approximately 13% and 14% in tensile strength and tensile modulus, respectively, for composites containing 1 wt.% ${\mathrm{TiO}}_{2}$, while 2 wt.% ${\mathrm{TiO}}_{2}$ led to improvements of approximately 19% and 40% in flexural strength and flexural modulus, respectively. These findings further support the role of ${\mathrm{TiO}}_{2}$ as an effective inorganic reinforcement for improving the mechanical response of polymer-based composites.

The development of nanofibers of polymers reinforced with several nanoparticles has gained interest in recent years because of the versatility that these nanofibers possess. Different advantages have been observed through several studies. For instance, electrospun nanofibers have a high surface area and tunable structural characteristics, which make them suitable for different applications such as composite materials fabrication [36], the development of sensors [37], or their use as specific adsorbents [38]. By combining polymer nanofibers with nanoparticles, a wide array of properties can be customized to meet the requirements of specific applications, enhancing their performance. Prior studies have examined low filler contents in polymer composites [39,40]. The three TiO_2_ contents evaluated here do not establish a percolation threshold.

The selection of low ${\mathrm{TiO}}_{2}$ nanoparticle concentrations was motivated by the need to balance mechanical reinforcement and nanoparticle dispersion within the polymer matrix. Previous studies have shown that low nanoparticle loadings can enhance the mechanical properties of polymer composites, whereas excessive concentrations may promote agglomeration, leading to stress concentration sites and reduced mechanical performance [41,42,43]. Therefore, low ${\mathrm{TiO}}_{2}$ contents were selected to examine electrospun fiber morphology and the tensile response of corresponding solution-cast membranes; nanoparticle dispersion was not directly quantified.

Recent reviews discuss sPEEK and composite membranes for proton-exchange membrane fuel cells [44]; however, no fuel-cell performance was measured in this study. As previously observed, different reinforcements have been added at different concentrations to sPEEK before and after electrospinning to obtain novel materials with improved and tailored properties. This study examines the morphology and FT-IR spectra of electrospun sPEEK/TiO_2_ fibers at low nanoparticle loadings. Tensile properties are measured separately on corresponding solution-cast membranes; those results are not direct measurements of fiber or electrospun-mat mechanics.

## 2. Materials and Methods

### 2.1. Raw Materials

Poly(ether ether ketone) (PEEK; ${\left(C_{19}H_{12}O_{3}\right)}_{n}$) powder with an average particle size of 80 μm and sulfuric acid ($H_{2}{\mathrm{SO}}_{4}$), used as the sulfonation medium, were obtained from Sigma-Aldrich (St. Louis, MO, USA). Commercial ${\mathrm{TiO}}_{2}$ nanoparticles (Jiangsu XFNANO, Nanjing, China; supplier-reported nominal particle size: 15–25 nm) were used as the inorganic filler. All materials were used as received.

### 2.2. Preparation of sPEEK-$TiO_{2}$ Nanofibers

To produce the different solutions, which will be subjected to the electrospinning process, PEEK polymer was first dried in a vacuum oven at a temperature of 100 °C for 24 h. Subsequently, three different amounts of PEEK (1.50, 1.62, and 1.75 g) were separately added to beakers containing 0.45 mL of deionized water and 15 mL of sulfuric acid ($H_{2}{\mathrm{SO}}_{4}$, 95% purity).The solutions were magnetically stirred at 110 °C for 3.5 h, until the solution turned reddish brown (sPEEK).

Several parameters in the electrospinning process influence the morphological and mechanical properties of the fibers. The most influential parameters include the distance from the capillary to the collector, the flow rate, and the voltage [36]. To determine the appropriate parameters for the electrospinning process, a Taguchi design of experiments ($L_{16}$) was developed by varying three different parameters: (1) distance between the tip of the needle (D) from 6 to 12 cm; (2) applied voltage (V) from 7 to 10 kV, and (3) the jet flow from (F) 0.25 to 1.75 mL/h (Table 1).

### 2.3. Characterization of Electrospun Fibers and Tensile Testing of Solution-Cast Membranes

**Figure 1:**
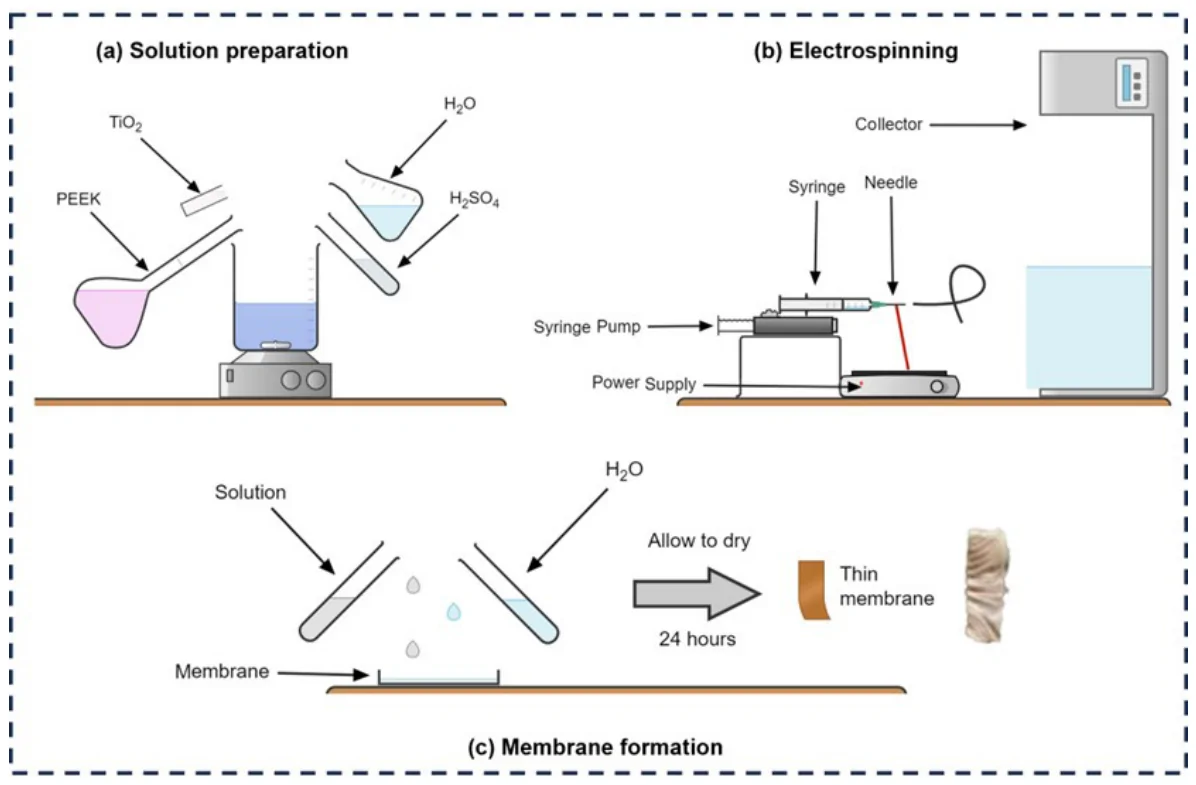
sPEEK-${\mathrm{TiO}}_{2}$ membranes preparation process and specimen geometry.

Once the combination of parameters has been determined to adequately obtain continuous fibers, three different quantities of ${\mathrm{TiO}}_{2}$ were added separately to the sPEEK solution to obtain a concentration of 0.04, 0.08, and 0.10 wt.% of the reinforcement. After the addition of ${\mathrm{TiO}}_{2}$, the resulting sPEEK/${\mathrm{TiO}}_{2}$ dispersions were magnetically stirred for an additional 2 h at 110 °C. Magnetic stirring was the only dispersion step applied. Each sPEEK-${\mathrm{TiO}}_{2}$ solution was loaded into a syringe equipped with a 25 G needle and connected to the corresponding syringe pump (Figure 1). The fibers were deposited onto a stationary flat collector plate located at the selected working distance. Following electrospinning, the fiber mats were vacuum-dried at 35 °C for 6 h to remove residual solvent before morphological and spectroscopic characterization. No post-spinning washing step was applied to the electrospun fibers. Neither the final pH nor the residual sulfate content of these specimens was measured; therefore, complete removal of residual sulfuric acid from the electrospun fibers could not be verified.

Fiber morphology was analyzed using a TESCAN MIRA3 scanning electron microscope (Tescan, Brno, Czech Republic) operated at 10 kV. The samples were mounted on conductive double-sided carbon tape and subsequently gold-coated at 15 mA and 80 mTorr for 60 s. Energy-dispersive X-ray spectroscopy (EDS) analysis was performed separately using a JEOL JSM-IT300 microscope equipped with a JED-2300 analysis system (JEOL Ltd., Tokyo, Japan). The analysis was conducted at an accelerating voltage of 30 kV, a probe current of 41.1 nA, and a live acquisition time of 452.36 s. The EDS spectrum was processed using a standardless ZAF method. In addition, ImageJ software (version 1.54p; National Institutes of Health, Bethesda, MD, USA) was used to obtain the average diameter of the electrospun fibers. Fourier-transform infrared spectroscopy (FT-IR) was performed to analyze the structure and composition of the fibers by the characteristic peaks of functional groups and chemical bonds present in the obtained spectra. FT-IR spectra were recorded within the 4000–400 cm^−1^ range by using a high-resolution spectrometer [38] and 25 cycles. Five different samples were tested through FTIR. The first sample only consisted of PEEK polymer, the second one was made from PEEK after the sulfonation process (sPEEK), and the rest of the specimens were made from sPEEK with different weight percentages of the ${\mathrm{TiO}}_{2}$ reinforcement (sPEEK-${\mathrm{TiO}}_{2}$).

**Figure 2:**
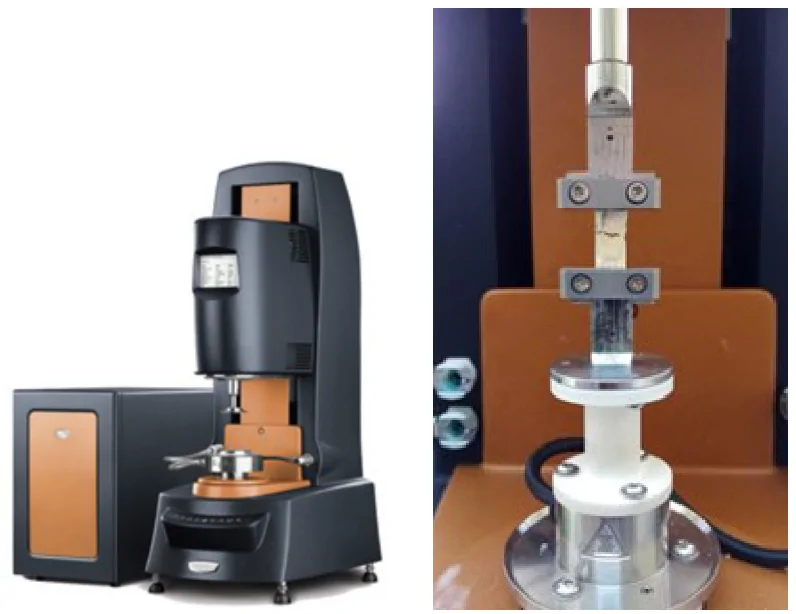
Tensile testing of sPEEK-TiO_2_ membranes: (**left**) DHR-2 rheometer; (**right**) specimen after fracture.

Since individual electrospun fibers could not be reliably handled for tensile testing, mechanical characterization was performed on solution-cast membranes prepared from the corresponding sPEEK and sPEEK/TiO_2_ formulations [45,46]. The solutions were cast in circular Petri dishes to form membranes, as shown in Figure 1c. The membranes were subsequently immersed in deionized water for 24 h, with the water replaced twice during this period [47]. Neither the final pH nor the residual sulfate content was measured; therefore, complete removal of residual sulfuric acid could not be confirmed. The membranes were then dried at room temperature for 48 h [48] and cut into rectangular specimens measuring 7.8×30 mm. Tensile tests were conducted using a DHR-2 rheometer (TA Instruments), as shown in Figure 2. The engineering stress–strain data were obtained using TRIOS software. Five specimens were tested per formulation.

Engineering stress and strain were calculated as $\sigma_{i}=F_{i}/\left(bt\right)$ and $\varepsilon_{i}=\Delta L_{i}/L_{0}$, respectively, where $F_{i}$ is the tensile force, *b* and *t* are the initial specimen width and thickness, respectively, and $L_{0}$ is the gauge length [49,50,51,52]. The apparent tensile modulus, $E_{\mathrm{app}}$, was determined independently for each specimen by ordinary-least-squares regression over the fixed strain interval 0.002≤ε≤0.020 [53,54]:

(1) $${\hat{\sigma }}_{i}=a+E_{\mathrm{app}}\varepsilon_{i}$$

(2) $$E_{\mathrm{app}}=\frac{\sum_{i}\left(\varepsilon_{i}-\bar{\varepsilon }\right)\left(\sigma_{i}-\bar{\sigma }\right)}{\sum_{i}{\left(\varepsilon_{i}-\bar{\varepsilon }\right)}^{2}}$$

where *a* is the fitted intercept, and $\bar{\varepsilon }$ and $\bar{\sigma }$ are the mean strain and stress values, respectively, within the fitted interval. Only data within 0.002≤ε≤0.020 were included in the modulus calculation; no smoothing or peak averaging was applied. The same strain interval and regression procedure were used for all specimens. The apparent tensile modulus, tensile strength, and elongation at fracture were reported as mean ± standard deviation (n=5).

## 3. Results and Discussion

### 3.1. Material Consolidation

Three different parameters, namely the applied external voltage, the distance between the needle tip and the collector, and the polymer jet flow rate, were systematically varied using a Taguchi design of experiments to identify the optimal electrospinning conditions. The selected levels for each parameter are summarized in Table 2, based on their reported influence on fiber formation and deposition.

The applied voltage plays a key role in initiating and sustaining the electrospinning process by inducing electrostatic forces that stretch the polymer jet. Moderate voltages promote fiber thinning, whereas excessive values may destabilize the jet and lead to bead formation [55,56]. In this study, the voltage was varied from 7 to 10 kV according to literature ranges and equipment limitations.

Similarly, the distance between the needle tip and the collector directly influences the flight time of the polymer jet, affecting solvent evaporation and fiber solidification. Insufficient distances may lead to incomplete solvent evaporation and bead formation, while excessive distances can result in discontinuous fibers [57,58]. Therefore, a range from 6 to 12 cm was considered.

The flow rate controls the volume of polymer solution supplied to the needle tip. Low flow rates may interrupt the jet, whereas high flow rates can lead to incomplete solvent evaporation and fiber defects [55,57]. Based on previous studies, the flow rate was varied from 0.25 to 1.75 mL/h.

The 16 conditions in Table 2 were evaluated. Fiber diameter was the numerical response for the L16 main-effects analysis; deposition quality and visible defects were also assessed qualitatively. Table 3 reports the supplied run-level mean diameters. A three-factor, four-level additive ANOVA of these 16 run means found an effect of needle-to-collector distance ($F_{3,6}=9.839$, p=0.00985), whereas voltage ($F_{3,6}=0.319$, p=0.812) and flow rate ($F_{3,6}=0.068$, p=0.975) were not significant at α=0.05. The corresponding descriptive shares of corrected total sum of squares were 80.47%, 2.61%, and 0.56%; the unexplained share was 16.36%. Because each condition contributes one run mean, the six residual degrees of freedom combine unmodelled interactions and other variation; the analysis does not independently estimate within-run measurement error. Run 8 (12 cm, 8 kV, 1.25 mL/h) had the lowest observed mean diameter (204.6695 nm) and was selected for subsequent fabrication based also on the observed uniformity of deposition, few visible defects, and alignment. The smallest factor-level mean for flow rate alone was at 0.75 mL/h, so selection of run 8 should not be interpreted as the combination of independently optimal levels for all three factors.

The results of the additive fixed-factor ANOVA are summarized in Table 4.

### 3.2. SEM and EDS Analysis

**Figure 3:**
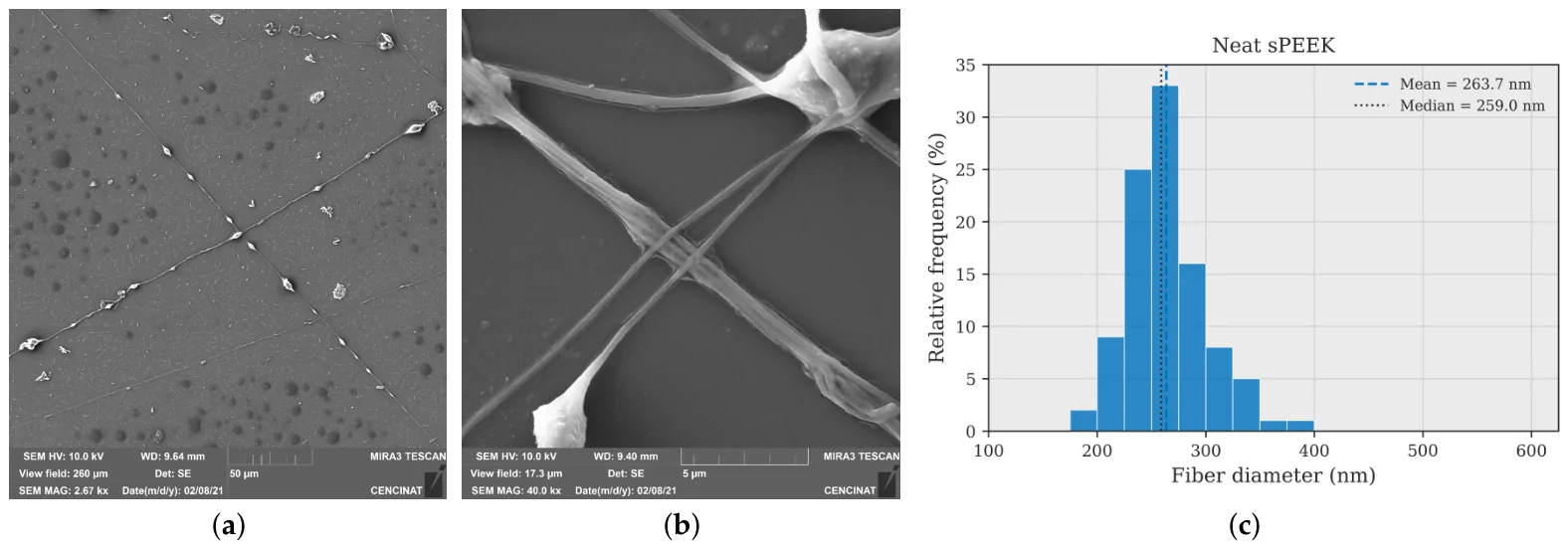
Representative SEM micrographs and fiber-diameter distribution of neat sPEEK fibers: (**a**) low magnification, (**b**) high magnification, and (**c**) diameter histogram.

Figure 3 presents representative SEM micrographs and the fiber-diameter distribution of neat sPEEK. The electrospinning technique can produce fibers of significant length, sometimes spanning several centimeters [56]. The fibers exhibited local diameter variations and occasional morphological irregularities. The mean and median diameters were 263.7 and 259.0 nm, respectively, with a coefficient of variation (CV) of 13.5%, indicating relatively limited dispersion among the analyzed regular fiber segments.

**Figure 4:**
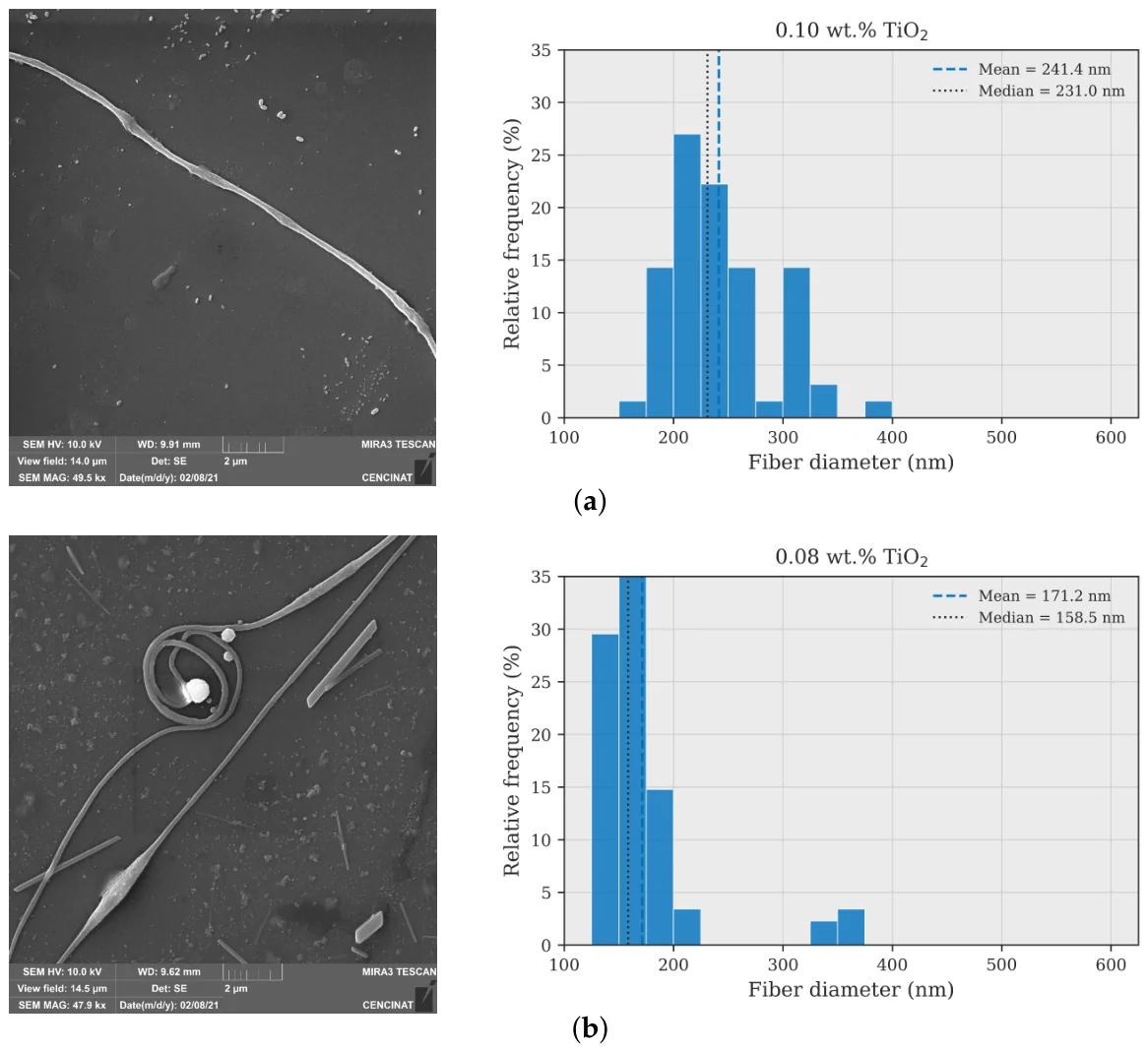

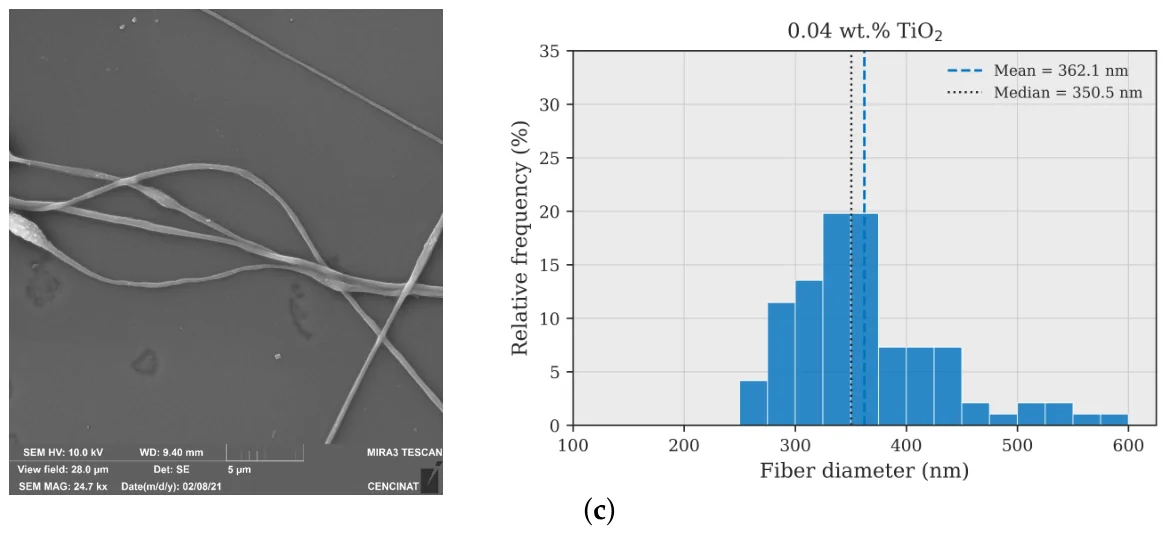
SEM micrographs (left) and corresponding fiber-diameter distributions (right) of electrospun sPEEK/TiO_2_ fibers containing (**a**) 0.10 wt.%, (**b**) 0.08 wt.%, and (**c**) 0.04 wt.% TiO_2_.

Figure 4 presents the corresponding SEM micrographs and diameter distributions of the sPEEK/TiO_2_ fibers. The mean diameters were 241.4, 171.2, and 362.1 nm for TiO_2_ contents of 0.10, 0.08, and 0.04 wt.%, respectively, while the corresponding median diameters were 231.0, 158.5, and 350.5 nm. The respective CV values were 20.1%, 28.1%, and 18.9%. Overall, fiber diameter and relative dispersion varied non-monotonically with TiO_2_ content. The 0.08 wt.% formulation exhibited the lowest mean diameter and the highest relative dispersion, whereas the 0.04 wt.% formulation exhibited the highest mean diameter. These observations could reflect changes in solution viscosity, electrical conductivity, or particle dispersion, but those variables were not measured for the individual formulations. No specific cause of the non-monotonic trend can therefore be definitively assigned or proven from the present SEM results alone.

**Figure 5:**
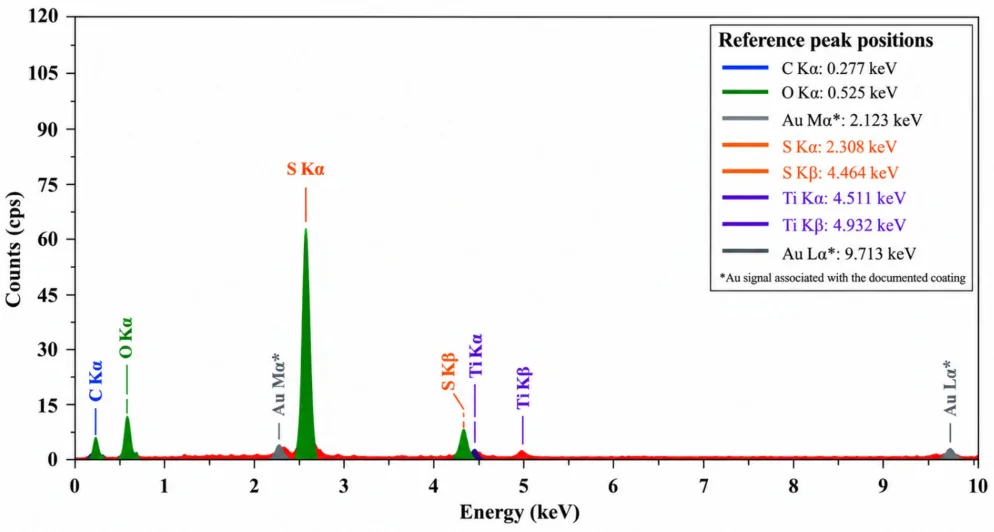
EDS spectrum of the electrospun sPEEK-0.10% ${\mathrm{TiO}}_{2}$ composite fibers showing the presence of C, O, S, and Ti.

The EDS spectrum of the sPEEK–0.10 wt.% TiO_2_ sample exhibited signals at energies consistent with the C Kα, O Kα, S Kα/Kβ, and Ti Kα/Kβ emission lines (Figure 5). The concurrent Ti Kα and Ti Kβ signals support the local presence of Ti-containing material in the analyzed region. Au-related signals were associated with the conductive coating. Because the spectrum was acquired from a coated fibrous specimen and processed using standardless ZAF analysis, the normalized elemental fractions were not considered representative of the bulk composite composition [59]. Accordingly, EDS was used only for qualitative elemental identification and not to determine the TiO_2_ concentration, polymer stoichiometry, or nanoparticle-distribution homogeneity.

### 3.3. FT-IR Spectroscopy Results

**Figure 6:**
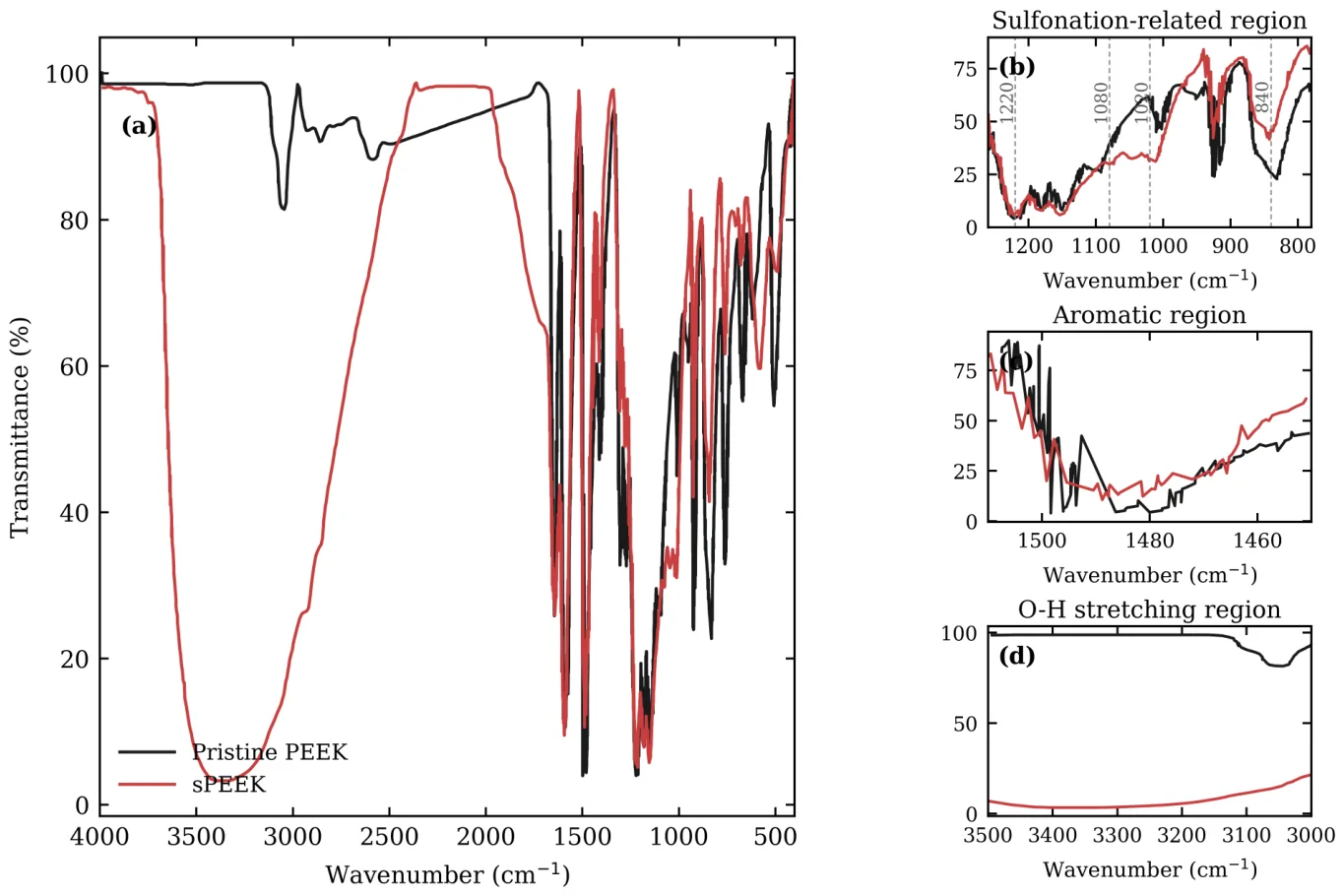
FT-IR spectra of pristine PEEK and sulfonated PEEK (sPEEK). (**a**) Overlaid spectra. (**b**) expanded 780–1260 cm^−1^ region showing the sulfonation-related spectral changes, (**c**) expanded 1450–1510 cm^−1^ aromatic region, and (**d**) expanded 3000–3500 cm^−1^ O–H stretching region.

Figure 6a displays the overlaid FT-IR spectra of pristine PEEK and sulfonated PEEK (sPEEK), with expanded views of the principal spectral regions affected by sulfonation in Figure 6b–d. Spectral changes are observed within the 780–1260 cm^−1^ range, particularly near 840, 1020, 1080, and 1220 cm^−1^. The features near 1020, 1080, and 1220 cm^−1^ are associated with vibrations involving sulfonic acid groups [60,61]. The change near 840 cm^−1^ is described as a sulfonation-related spectral modification because this region may also contain contributions from the PEEK fingerprint. Within the aromatic region (1450–1510 cm^−1^), the band near 1487 cm^−1^ in pristine PEEK is resolved into features near 1489 and 1471 cm^−1^ after sulfonation [62]. The broad absorption between 3000 and 3500 cm^−1^ is attributed to O–H stretching involving sulfonic acid groups and hydrogen-bonded species [20,60]. Collectively, these spectral changes provide qualitative evidence that PEEK sulfonation occurred.

Although the FT-IR bands assigned to sulfonic acid groups confirm that sulfonation occurred, they do not enable quantitative determination of the degree of sulfonation (DS). Because DS can affect the hydrophilicity, water uptake, swelling, chain mobility, and mechanical properties of sPEEK membranes, the results reported here are specific to the sulfonation conditions employed [63]. Accordingly, potential relationships between DS, fiber morphology, and sPEEK–${\mathrm{TiO}}_{2}$ interfacial interactions could not be assessed.

**Figure 7:**
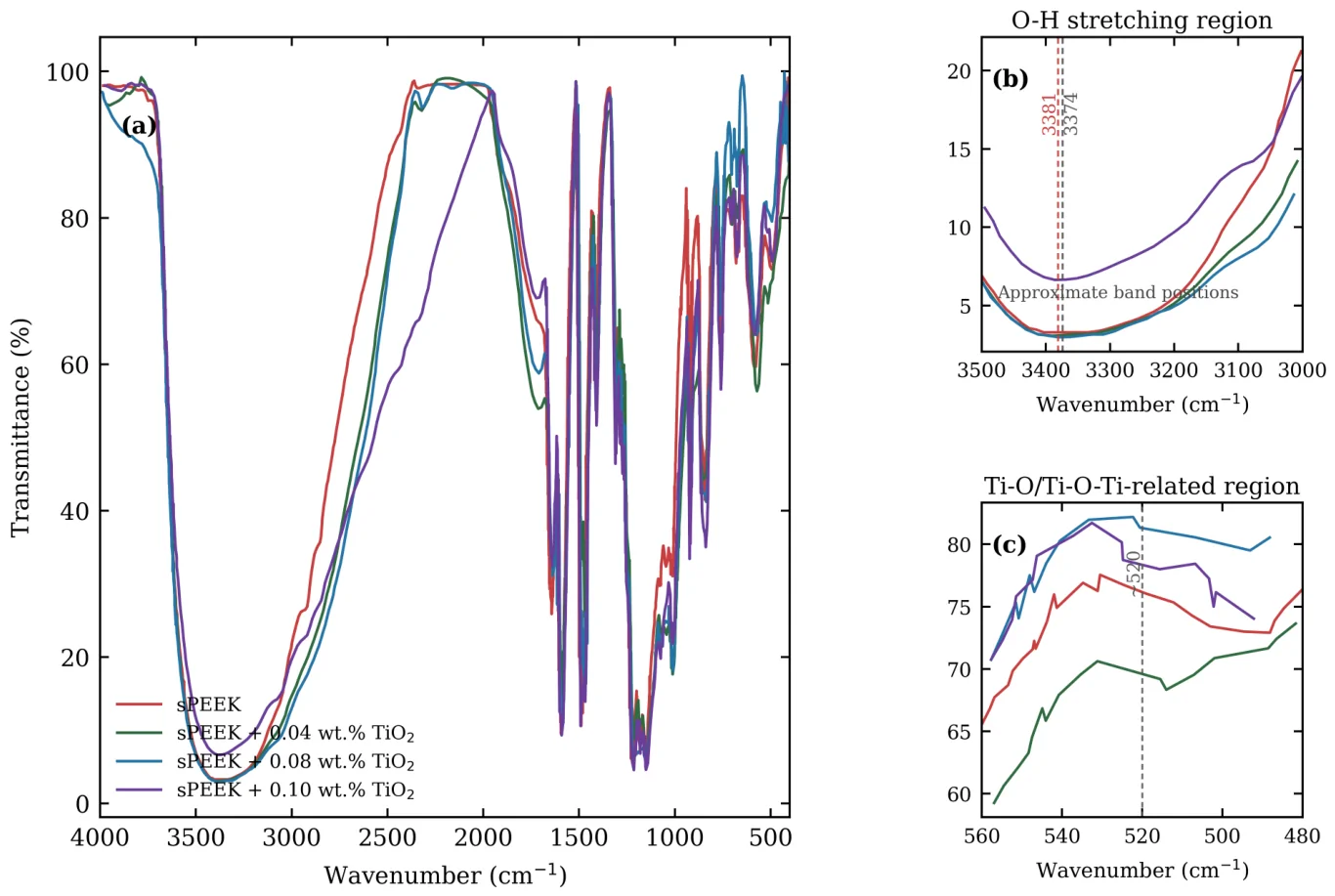
FT-IR spectra of sPEEK/TiO_2_ composite fibers with different TiO_2_ contents (0.04, 0.08, and 0.10 wt%). (**a**) Full spectra. (**b**) expanded O–H stretching region between 3000 and 3500 cm^−1^, showing the subtle variation from approximately 3381 to 3374 cm^−1^; and (**c**) expanded 560–480 cm^−1^ low-wavenumber region associated with possible Ti–O/Ti–O–Ti contributions.

Figure 7 presents the FT-IR spectra of neat sPEEK and sPEEK/TiO_2_ composite fibers containing 0.04, 0.08, and 0.10 wt.% TiO_2_, together with expanded views of the O–H stretching and low-wavenumber regions. As shown in Figure 7b, the broad O–H absorption exhibits subtle variations in position and shape in the region centered at approximately 3381–3374 cm^−1^. Considering the breadth of this absorption and the small magnitude of the variation, these differences cannot be regarded as a distinct diagnostic shift. They may instead reflect a modification of the local hydrogen-bonding environment. Interactions involving the sulfonic acid groups of sPEEK and surface sites on TiO_2_ have been proposed for related sPEEK/TiO_2_ systems [61]; thus, such interactions constitute a plausible interpretation of the present observations. Nevertheless, this assignment remains tentative because the surface chemistry of the TiO_2_ employed in this study was not independently characterized.

Changes are also observed near 520 cm^−1^ in Figure 7c, within a low-wavenumber region where Ti–O-related lattice vibrations may contribute. Because the TiO_2_ concentrations are low and this region overlaps with vibrations of the polymer matrix, no clearly isolated Ti–O or Ti–O–Ti band can be resolved. Accordingly, the observed variations are considered compatible with contributions from TiO_2_-related vibrational modes, but they cannot be assigned unambiguously. EDS therefore provides complementary evidence for the presence of Ti in the analyzed fibers; however, it does not independently establish its chemical state, confirm a specific TiO_2_ phase, or demonstrate homogeneous nanoparticle dispersion.

Interactions between the sulfonic acid groups of sPEEK and inorganic oxide surfaces have been reported in related hybrid membrane systems [61,64]. In the present study, the subtle variations observed in the O–H stretching and low-wavenumber regions may reflect changes in the local hydrogen-bonding environment after TiO_2_ incorporation. These potential interfacial interactions could influence load transfer between the polymer matrix and the inorganic phase and may therefore contribute to the observed mechanical-property trends. This interpretation remains tentative because the FT-IR results provide indirect evidence and do not independently resolve the molecular structure of the sPEEK–TiO_2_ interface or establish a direct causal relationship with the mechanical response.

### 3.4. Mechanical Characterization

**Figure 8:**
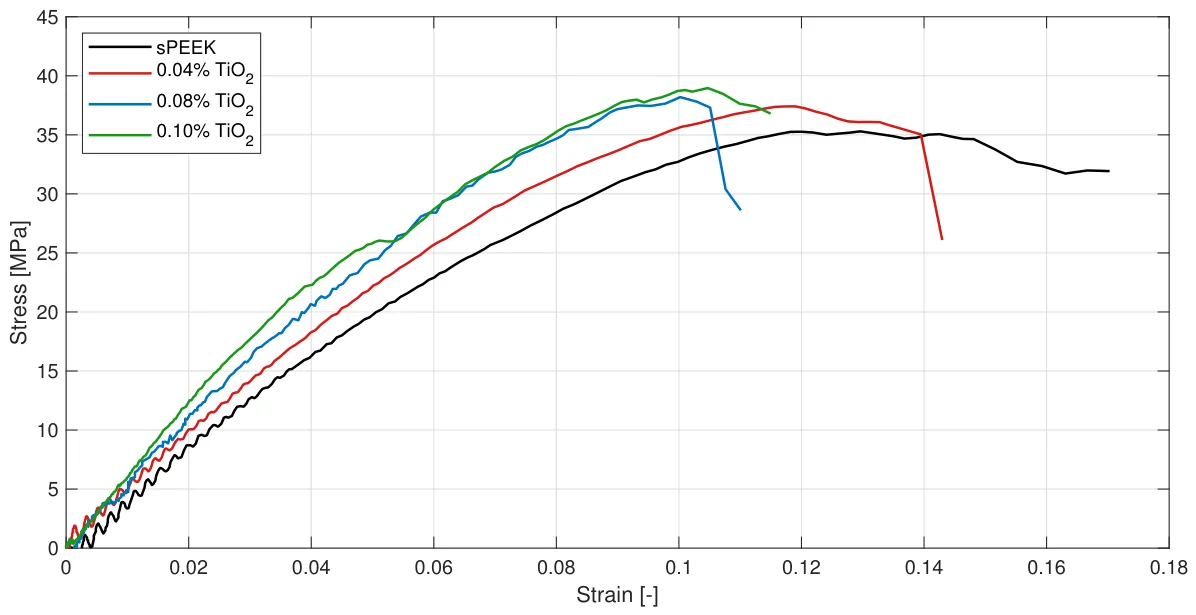
Representative engineering stress–strain curves of sPEEK and sPEEK/TiO_2_ specimens. The apparent tensile modulus was determined by linear regression over 0.002≤ε≤0.020; the measured curves were not smoothed.

**Figure 9:**
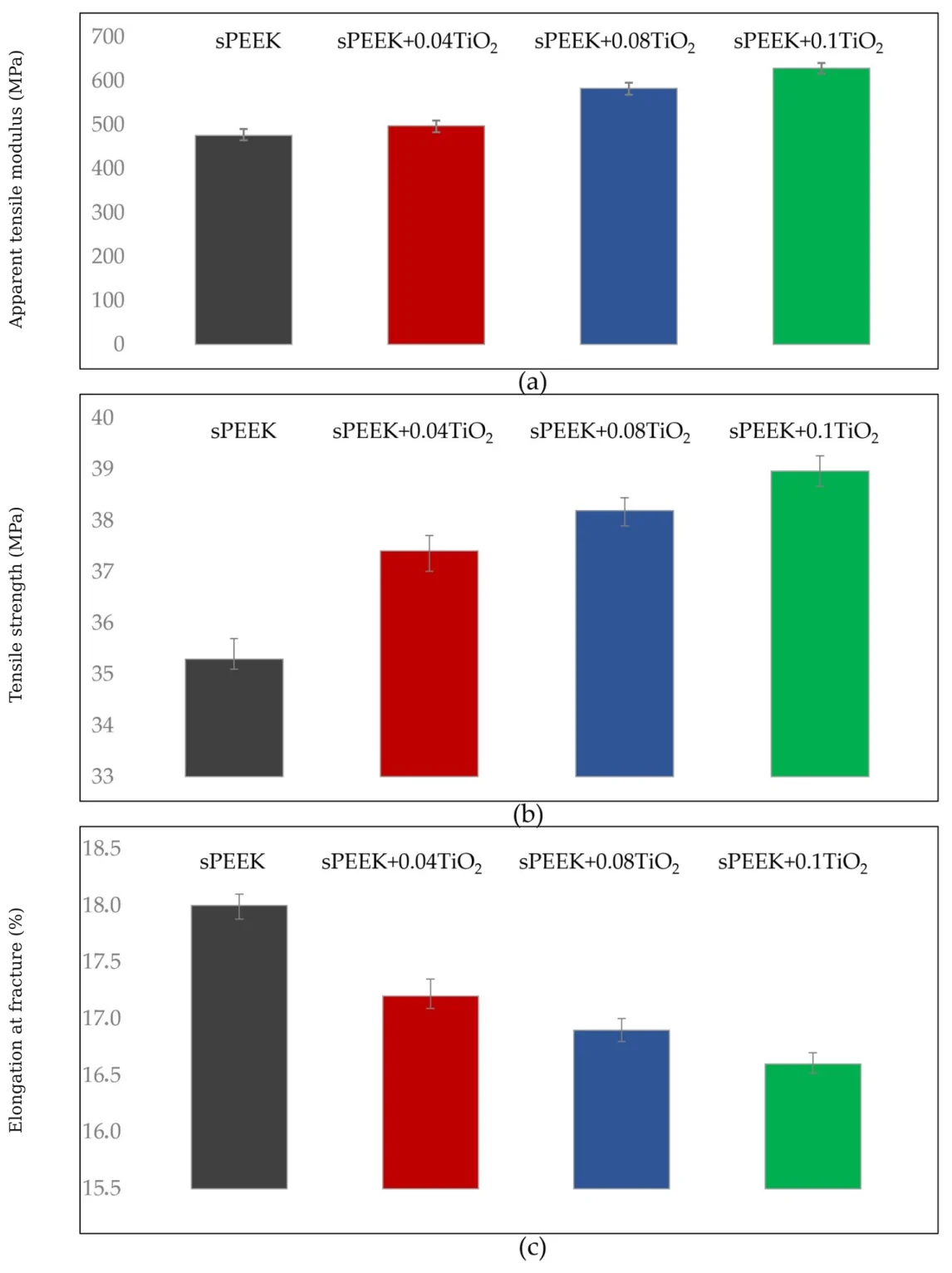
Mean mechanical properties of neat sPEEK and sPEEK/TiO_2_ formulations: (**a**) apparent elastic modulus, (**b**) apparent tensile strength, and (**c**) elongation at fracture. Error bars represent one standard deviation (n=5).

Figure 8 presents representative stress–strain curves for the solution-cast sPEEK and sPEEK/TiO_2_ membrane specimens. Minor initial oscillations may reflect specimen seating or grip-related effects; however, their origin remains unconfirmed because slippage was not independently monitored. Only data within the fixed strain interval 0.002≤ε≤0.020 were included in the regression used to determine the apparent tensile modulus; no smoothing or peak averaging was applied. Table 5 reports the tensile properties as mean ± standard deviation (n=5), whereas Figure 9 compares the corresponding mean values.

Figure 9 compares the mean apparent tensile modulus, tensile strength, and elongation at fracture. Neat sPEEK membranes exhibited values of 476±15 MPa, 35.30±0.40 MPa, and 18.0±0.10%, respectively (Table 5).

Relative to neat sPEEK, the 0.04, 0.08, and 0.10 wt.% TiO_2_ formulations showed increases in mean apparent tensile modulus of approximately 5%, 22%, and 32%, respectively. The corresponding increases in mean tensile strength were approximately 6%, 8%, and 10%, whereas mean elongation at fracture decreased by approximately 4%, 6%, and 8%. These differences describe the measured sample means; no specimen-level inferential comparison was carried out here, and statistical significance of individual pairwise differences has not been established. Similar filler-dependent trends in tensile behavior have been reported for nanoparticle-modified polymer membranes, although direct quantitative comparison is limited by differences in polymer matrix, filler chemistry, reinforcement content, and processing conditions [65,66,67].

The increases in mean apparent tensile modulus and tensile strength may reflect interfacial interactions between TiO_2_ surface hydroxyl groups and the sulfonic acid groups (SO_3_H) of sPEEK, consistent with the small FT-IR shift in the O–H region. Such interactions could restrict segmental mobility and improve stress transfer [61,68,69]. The concurrent reduction in elongation may also involve local stress concentrations associated with rigid inclusions, interfacial heterogeneity, or particle aggregates [26,70]. These mechanisms remain hypotheses because particle dispersion and fracture processes were not directly resolved.

Further characterization by X-ray photoelectron spectroscopy, high-resolution transmission electron microscopy with elemental mapping, and dynamic mechanical analysis would be required to directly examine the proposed interfacial interactions and their effect on polymer-chain mobility. In addition, post-fracture microscopy would help assess whether microvoiding, microcracking, particle–matrix debonding, or other localized damage mechanisms contributed to the reduction in elongation at fracture. Appropriate fracture-mechanics testing would also be required before drawing conclusions regarding the fracture toughness of the sPEEK/TiO_2_ specimens.

The membrane wash was not checked by measuring the final rinse pH or residual sulfate. Residual acid, if present, could affect membrane behavior and confound attribution of the tensile differences solely to TiO_2_. Its amount and effect cannot be inferred from the present measurements. Future work should verify washing and residual sulfate under the same conditions for all formulations.

The present study was limited to the initial morphological, chemical, and tensile response of the materials after fabrication. Long-term stability and durability were not evaluated. Consequently, the retention of fiber morphology, chemical structure, and mechanical properties during prolonged exposure to humidity, elevated temperature, cyclic mechanical loading, or chemically aggressive environments remains to be established. The current results should therefore be interpreted as an initial characterization and should not be extrapolated to long-term service performance. Future work should include accelerated aging under controlled environmental conditions, cyclic loading, and post-aging SEM, FT-IR, and tensile characterization to quantify property retention and identify possible degradation mechanisms.

## 4. Conclusions

This work characterizes electrospun sPEEK/TiO_2_ fibers and separately evaluates the tensile properties of corresponding solution-cast membranes. The principal findings are:

- Across the 16 supplied run means, distance showed the strongest main effect on fiber diameter ($F_{3,6}=9.839$, p=0.00985). Run 8 (12 cm, 8 kV, and 1.25 mL/h) yielded the lowest observed mean diameter (204.6695 nm) and was selected with qualitative deposition criteria.
- A correct sulfonation process was confirmed by means of FT-IR due to the presence of peaks at approximately 840, 1020, 1080, and 1220 cm^−1^ corresponding to S-O stretching vibration, S=O stretch, symmetric stretching vibration of aromatic ${\mathrm{SO}}_{3}H$, asymmetric stretching vibration of O=S=O. In addition, it was observed that the peak at 1487 cm^−1^ found in the PEEK spectrum was divided into different peaks at 1489 and 1471 cm^−1^ produced by substituting different aromatic rings with sulfonic acid groups.
- FT-IR spectra suggested possible interactions between the sulfonated polymer and the reinforcement. The peak found in sPEEK at approximately 3381 cm^−1^, corresponding to the hydroxyl group, slightly shifted toward lower wavenumbers, which may be associated with hydrogen-bonding interactions involving hydroxyl groups at the ${\mathrm{TiO}}_{2}$ surface. Moreover, additional bands in the low-wavenumber region were associated with Ti–O–Ti vibrations. These results should be interpreted as indirect evidence of possible polymer–particle interactions rather than definitive confirmation of chemical bonding or nanoparticle dispersion.
- Within the evaluated range, increasing TiO_2_ content was associated with higher mean apparent tensile modulus and tensile strength and lower mean elongation at fracture. Relative to neat sPEEK, the 0.10 wt.% TiO_2_ formulation showed increases of approximately 32% in mean apparent tensile modulus and 10% in mean tensile strength, together with an approximately 8% decrease in mean elongation at fracture. These differences are descriptive; specimen-level data and a defined inferential analysis are needed to establish pairwise statistical significance. A percolation threshold was not established, and membrane tensile properties cannot be assigned to individual electrospun fibers.
- Long-term stability and durability were outside the scope of the present study. Therefore, accelerated aging and post-aging morphological, chemical, and mechanical characterization are required before the long-term service performance of the sPEEK/TiO_2_ materials can be established.

## Figures and Tables

**Table 1:** Experimental variables employed for the electrospinning of the fibers.

Parameters	Symbol	Min	Max
Distance (cm)	D	6	12
Voltage (kV)	V	7	10
Jet Flow (mL/h)	F	0.25	1.75

**Table 2:** Taguchi L16 design used for electrospinning and qualitative observations of fiber deposition.

Run	Distance (cm)	Voltage (kV)	Flow Rate (mL/h)	Observation
1	6	7	0.25	Minimal fiber deposition.
2	8	7	0.75	Irregular deposition, presence of lumps.
3	10	7	1.25	Irregular deposition, fibers with lumps.
4	12	7	1.75	Fibers reached the collector, acceptable deposition.
5	6	8	0.75	Irregular deposition, high lump content.
6	8	8	0.25	Irregular deposition, high lump content.
7	10	8	1.75	Irregular deposition, clumpy fibers.
8	12	8	1.25	Uniform deposition, few defects, good alignment.
9	6	9	1.25	Good deposition, short fibers.
10	8	9	1.75	Good deposition, few defects.
11	10	9	0.25	Irregular deposition, incomplete jet.
12	12	9	0.75	Fine fibers obtained, slight beading.
13	6	10	1.75	Moderate defects.
14	8	10	1.25	Good deposition, proper alignment.
15	10	10	0.75	Good deposition, slight bead formation.
16	12	10	0.25	Excessive dripping, unstable jet.

**Table 3:** Run-level mean fiber diameters supplied for the L16 electrospinning design. The S/N values in the supplied worksheet were not used in the ANOVA because their calculation needs reconciliation with the displayed rounded diameters.

Run	Distance (cm)	Voltage (kV)	Flow (mL/h)	Mean Diameter (nm)
1	6	7	0.25	224.6254
2	8	7	0.75	223.3924
3	10	7	1.25	224.0743
4	12	7	1.75	211.8033
5	6	8	0.75	219.9566
6	8	8	0.25	220.6022
7	10	8	1.75	228.2792
8	12	8	1.25	204.6695
9	6	9	1.25	228.5729
10	8	9	1.75	213.4348
11	10	9	0.25	232.5302
12	12	9	0.75	211.4352
13	6	10	1.75	225.1772
14	8	10	1.25	222.3646
15	10	10	0.75	222.9234
16	12	10	0.25	206.1588

**Table 4:** Additive fixed-factor ANOVA of the 16 run-level mean fiber diameters. Contributions are descriptive shares of corrected total SS; the residual includes unmodelled interactions.

Source	df	SS (nm^2^)	MS (nm^2^)	*F*	*p*	Contribution (%)
Distance	3	804.8005	268.2668	9.8387	0.00985	80.47
Voltage	3	26.1005	8.7002	0.3191	0.81177	2.61
Flow rate	3	5.6002	1.8667	0.0685	0.97469	0.56
Residual	6	163.5992	27.2665	–	–	16.36
Total	15	1000.1004	–	–	–	100.00

**Table 5:** Mechanical properties of neat sPEEK and sPEEK/TiO_2_ specimens. Values are reported as mean ± standard deviation (n=5).

Formulation	Apparent Tensile Modulus, $E_{\mathrm{app}}$ (MPa)	Tensile Strength (MPa)	Elongation at Fracture (%)
sPEEK	476±15	35.30±0.40	18.0±0.10
sPEEK + 0.04 wt.% TiO_2_	498±12	37.41±0.30	17.2±0.15
sPEEK + 0.08 wt.% TiO_2_	583±13	38.20±0.25	16.9±0.10
sPEEK + 0.10 wt.% TiO_2_	629±12	38.97±0.30	16.6±0.10

*Note: *$E_{\mathrm{app}}$ was obtained independently for each specimen by ordinary-least-squares regression over 0.002≤ε≤0.020.

## Data Availability

Data are available upon request from the corresponding authors.
